# Mutations in the Hedgehog Pathway Genes *SMO* and *PTCH1* in Human Gastric Tumors

**DOI:** 10.1371/journal.pone.0054415

**Published:** 2013-01-18

**Authors:** Xi-De Wang, Hector Inzunza, Han Chang, Zhenhao Qi, Beihong Hu, Daniel Malone, John Cogswell

**Affiliations:** Bristol-Myers Squibb, Princeton, New Jersey, United States of America; Indiana University School of Medicine, United States of America

## Abstract

The causal role of the hedgehog pathway in cancer has been best documented in basal cell carcinoma of the skin. To assess potential DNA alterations of the hedgehog pathway in gastric cancer, we sequenced *SMO* and *PTCH1* genes in a set of 39 gastric tumors. Tumors were classified by histology based on the Lauren classification and Sanger sequencing was performed to obtain full length coding sequences. Genomic instability was evident in these tumors as a number of silent or missense mutations were found. In addition to those that are potential germline polymorphisms, we found three *SMO* missense mutations, and one *PTCH1* frameshift mutation that are novel and have not been documented in basal cell carcinoma. Mutations were found in both intestinal and diffuse type gastric tumors as well as in tumors that exhibit both intestinal and diffuse features. mRNA expression of hedgehog pathway genes was also examined and their levels do not indicate unequivocal higher pathway activity in tumors with mutations than those without. In summary, *SMO* and/or *PTCH1* mutations are present at low frequency in different histologic subtypes of gastric tumors and these do not appear to be driver mutations.

## Introduction

The hedgehog signaling pathway is critical for embryonic development. Its role in cancer was first revealed when the linkage of the human patched 1 (*PTCH1*) gene was established with the basal cell nevus syndrome (BCNS or Gorlin syndrome), a rare hereditary disorder characterized by multiple basal cell carcinomas (BCC) and predisposition to medulloblastomas [1], [2]. High frequency of hedgehog pathway mutations was also found in sporadic BCCs, where about 90% have loss-of function mutations in *PTCH1* and about 10% have activating mutations in the smoothened gene (*SMO* ) [3], [4], [5]. This causal relationship guided the successful development of vismodegib (GDC-0449), a small molecule antagonist targeting the hedgehog pathway that was recently approved for the treatment of metastatic or recurrent locally advanced BCC [6].

Gastric cancer is the fourth most commonly diagnosed cancer and the second most common cause of cancer death worldwide [7]. Histologically, gastric cancer can be classified into two major types, intestinal and diffuse [8]. While the intestinal type is characterized by clustered, glandular-like and differentiated histology, the diffuse type is scattered, infiltrative and poorly differentiated. Expression of hedgehog pathway genes has been documented in gastric cancer cell lines and gastric adenocarcinomas [9], [10]. Furthermore, it has been reported that diffuse gastric cancer exhibits higher level expression of hedgehog pathway genes (including ligands, receptors and downstream effectors) than intestinal metaplasia or intestinal gastric cancer [11]. Overexpression of pathway signaling components is thought to be one major mechanism of hedgehog pathway activation in tumors other than BCCs [12].

DNA alterations including chromosome rearrangements, gene amplifications, and somatic mutations have been demonstrated to be salient indicators of tumor addiction to oncogene function and efficacy predictors for a number of targeted cancer therapeutics such as imatinib, trastuzumab, and erlotinib in chronic myeloid leukemia (CML), breast cancer, or lung cancer respectively [13]. Development of vismodegib for use in basal cell carcinoma has apparently followed a similar paradigm. Identification of novel DNA level alterations in a common set of genes after beneficial tumor response to initial therapy not only supports the causal relationship between driver gene mutations and disease, but may also guide the development of second-generation therapies overcoming resistance. One example is the evolution of three generations of tyrosine kinase inhibitors targeting BCR-ABL in CML [14]. Interestingly and perhaps not surprisingly, in the early clinical testing of vismodegib [15], a medulloblastoma patient who presented with widespread metastatic disease and had a *PTCH1* mutation responded rapidly and achieved dramatic tumor regression. However, this patient quickly developed resistance to the therapy and presented with a previously unidentified mutation in *SMO* that alters responsiveness to the hedgehog pathway inhibitor [16]. To gain insight into the causal role of the hedgehog pathway in gastric cancer, we sequenced the full length coding region of both *SMO* and *PTCH1* genes in a panel of 39 gastric tumors of different histologic subtypes. We also assessed expression-level consequence of the detected mutations by examining pathway gene expression in the mutated tumors.

## Materials and Methods

### Tumor Samples and Histologic Analysis

Thirty nine gastric tumor samples with original histopathologic assessment information were acquired from a commercial source (Asterand, Detroit, MI). Samples were selected based on >70% tumor content by hematoxylin and eosin (H&E) staining, >6 RIN (RNA intact numeric score) of the matched mRNA preparation, and the availability of frozen tissue blocks. All samples were derived from patients of Caucasian origin. The histology of these was re-evaluated using the actual formalin-fixed, paraffin-embedded (FFPE) blocks obtained using the Lauren classification. Images were captured under a NIKON ECLIPSE E800 microscope with an Advanced Imaging Concepts AC500CS CMOS camera. Unique identities of this set of tumors were confirmed using all annotations derived from the study, including histology, gene mutations, and global mRNA expression.

### DNA Isolation, Mutation and mRNA Analyses

Frozen tumor specimens were processed using QIAamp DNA Mini Kit per manufacturers’ manual (Qiagen, Valencia, CA). DNA was quantified using Nanodrop (Thermo Fischer, Wilmington, DE). All samples yielded at least 2.5 µg DNA for sequencing.

*SMO* (NM_005631) and *PTCH1* (NM_000264.3) coding sequences were determined using polymerase chain reaction (PCR) and Sanger sequencing on genomic DNA purified from freshly frozen tumor samples. Twelve and 25 sets of primers (Table S1) were used for *SMO* and *PTCH1*, respectively. Primer sets were designed to cover the entire coding sequences plus where applicable a few nucleotides into the intron sequences on both ends. In two cases where PCR primers did not generate acceptable reads, sequencing primers annealing to inner sequences were designed to achieve high quality data. Primer extension sequencing was performed by GENEWIZ, Inc (South Plainfield, NJ) using Applied Biosystems BigDye version 3.1. Both forward and reverse strands were sequenced. The reactions were processed using an Applied Biosystem’s 3730xl DNA Analyzer, and the sequencing data were analyzed with Lasergene SeqMan (DNASTAR, Madison, WI). All initial mutation calls were confirmed on Mutation Surveyor (SoftGenetics, State College, PA) by a separate researcher.

Microarray analysis was performed using HG-U133A_PM chips following standard Affymetrix procedures (Affymetrix, Santa Clara, CA). Raw data was processed with RMA algorithm, and expression value in log scale was visualized according to gene mutation status on Partek Genomic Suites (Partek, Saint Louis, MO).

Real-time quantitative RT-PCR (qRT-PCR) was performed with ABI ViiA™ 7 Real-Time PCR System (384-well module) (Applied Biosystems, Foster City, CA). Two normal gastric tissue total RNA samples were obtained commercially (Biochain, Newark, CA and Clontech, Mountain View, CA). Total RNA was quality checked with Agilent 2100 Bioanalyzer (Agilent, Santa Clara, CA) and quantified using NanoDrop ND-1000 (NanoDrop Technologies, Wilmington, DE). cDNA was synthesized with ABI High Capacity RNA to cDNA kit (Applied Biosystems, Foster City, CA). The following primer-probe sets were used: GLI1-Hs00171790_m1, GLI2_Hs01119974_m1, GLI3-Hs00609233_m1, PTCH1-Hs00181117_m1, PTCH2_Hs01085642_m1, and house-keeping gene GUSB_Hs99999908_m1 (Applied Biosystems, Foster City, CA). Gene expression levels were calculated using equation 2^−deltaCt^.

## Results and Discussion

### Tumor Histology

**Figure 1:**
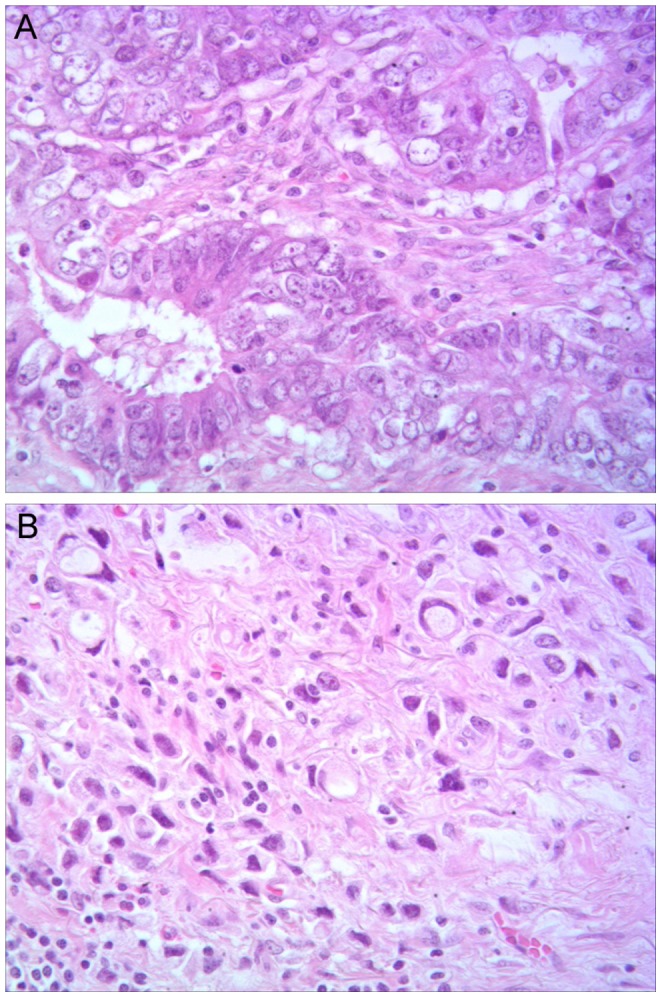
Representative H&E staining images for histopathologic assessment of gastric tumors. (A) A moderately differentiated intestinal type adenocarcinoma. Note the glandular pattern of neoplastic growth, tumor cells with scant cytoplasm and conspicuous nucleoli, and the fibrous tissue associated with lymphocytic infiltrate between glands. (B) An adenocarcinoma of diffuse type. Note single cells with signet ring features infiltrating the gastric wall. (images are at 40× magnification).

The tumor samples analyzed in this study were collected from patients with mainly stage I to stage III localized gastric cancers that had few or no lymph node metastases. To investigate whether potential mutations occur preferentially in specific histologic subtypes, we first performed H & E staining to classify tumors according to the Lauren classification. We identified 10 as intestinal and 21 as diffuse type gastric tumors. Examples of these two types of histology are shown in Fig. 1. Interestingly, we also found 7 gastric tumors that show features of both intestinal and diffuse subtypes in the same specimen, typically with less cohesive infiltrating cells (diffuse type) separated from glandular malignant cells (intestinal type) by thick fibrous tissues (data not shown). Subtype of one tumor could not be confirmed due to insufficient tumor tissue sample on the slide.

### SMO Mutations

**Figure 2:**
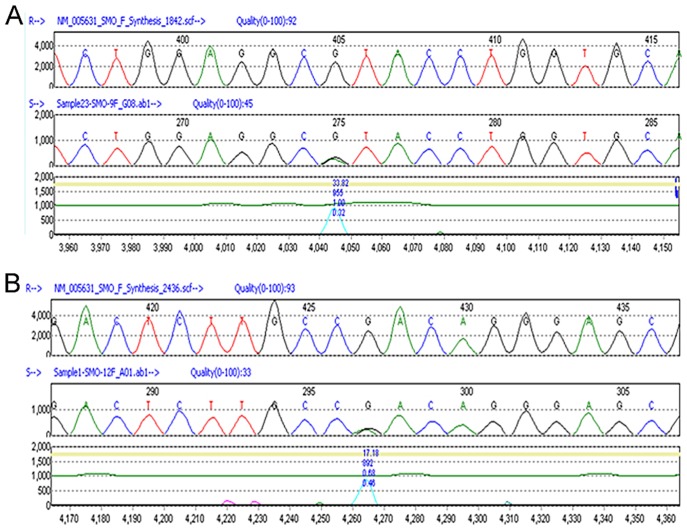
Examples of novel *SMO* missense mutations. (A) SMO R547H mutation found in an intestinal type gastric tumor (sample 23). (B) SMO R726Q mutation found in an intestinal type gastric tumor (sample 1).

A number of coding variants were identified in the *SMO* gene. The most common variant, at nucleotide position G1164, was found in 10 patients (G1164C, with G/C alleles) while another 28 patients exhibited C/C alleles. This nucleotide change does not result in protein amino acid change (at *p.*G388). Several other silent mutations/polymorphisms (c.C384T, c.C717T, c.C1182T, c.G1137A, c.T1722C, and c.G2052A) were identified in 7 tumors (data not shown), suggesting genomic instability in these tumors. Missense mutations, grouped by histologic subtypes, are summarized in Table 1. The chance of these missense mutations representing potential germline polymorphisms was investigated by mining of published SNP database curated by National Center for Biotechnology Information (NCBI) and The 1000 Genomes Project. The p.D25G, p. V270I, p.R168H variants were found to be polymorphisms. Three others mutations, including p.17insL, p.R547H, p.R726Q, were determined to be novel mutations and their functional implication is unknown. Interestingly, one intestinal type tumor had three different mutations. It appears that all three subtypes of gastric tumor exhibit novel mutations in the *SMO* gene and the mutation frequency is around 10% (4/39). Sequencing traces for p.R547H in sample 23 (A) and for p.R726Q in sample 1 (B) are shown in Fig. 2.

**Table:** **Table 1.** *SMO* and *PTCH1* Mutations in 39 Gastric Tumors.

Gene	Histology	Sample ID	Exon	DNA	Protein	Class	Function
*SMO*	Diffuse	2	1	c.A74G	p.D25G	Missense	Polymorphism
	Diffuse	14	1	c.48insGCT	p.17insL	Insertion	Unknown
	Diffuse	21	1	c.A74G	p.D25G	Missense	Polymorphism
	Diffuse	24	4	c.G808A	p.V270I	Missense	Polymorphism
	Intestinal	1	12	c.G2177A	p.R726Q	Missense	Unknown
	Intestinal	8	2	c.G503A	p.R168H	Missense	Polymorphism
	Intestinal	13	1	c.A74G	p.D25G	Missense	Polymorphism
	Intestinal	23	1	c.A74G	p.D25G	Missense	Polymorphism
		23	9	c.G1640A	p.R547H	Missense	Unknown
		23	12	c.G2177A	p.R726Q	Missense	Unknown
	Mixed	3	1	c.48insGCT	p.17insL	Insertion	Unknown
*PTCH1*	Intestinal	11	23	c.C3921del	p. R1307fs	Frameshift	Loss of Function
	Various	20 samples	23	c.C3944T	p. P1315L	Missense	Polymorphism

### PTCH1 Mutations

**Figure 3:**
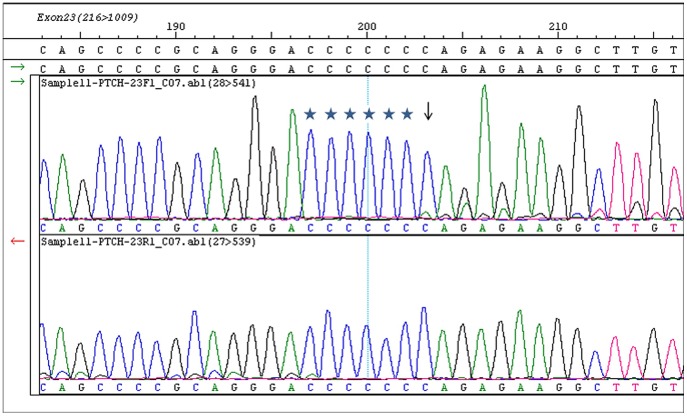
*PTCH1* frameshift mutation in an intestinal type gastric tumor. Shown are forward (top panel) and reverse sequencing (bottom panel) around c. C3921 region for sample 11. Arrow indicates position 3921 where the first shifted nucleotide was detected. Note that in this situation where a stretch of 7 cytosines are present the deletion could also have happened at any of the six preceding nucleotide positions (asterisk). Reverse trace confirmed the frameshift mutation (bottom panel). Under similar reaction conditions other tumors are wild type in this region.

The types of mutations detected in *PTCH1* appeared much more limited than those found in *SMO*. Only two types of variants were identified in this panel of tumors. The most evident is the c.C3944T single nucleotide change in exon 23 that results in a p. P1315L amino acid change. This variant, identified in 20 tumors (∼50% frequency), very likely represents a polymorphism, a hypothesis in agreement with previous studies [17], [18]. The second alteration identified is a frameshift mutation in an intestinal type tumor. As shown in Fig. 3, deletion of a cytosine at position 3921 caused a single nucleotide shift in coding sequence. The height of the shifted peak appears approximately 5–10% of the peak relative to the wild type nucleotide, suggesting that the observed deletion represents a localized mutation, or perhaps a lower than expected tumor contribution to the tissue specimen used for sequencing. Technically, the observed frameshift mutation could have arisen from deletion of any of the 6 nucleotides 5′ of position 3921, as the shifted peaks would be indistinguishable from the same nucleotide (all cytosine) present one nucleotide ahead of the deletion in the wild type sequence. It should be noted that this observation is unlikely to be due to DNA polymerase slippage during sequencing reaction, as all other samples showed clean wild type sequences in this region. Thus, loss of function mutation in *PTCH1* is likely present in this tumor.

### Pathway Activation Assessment by Down-stream Gene Expression

**Figure 4:**
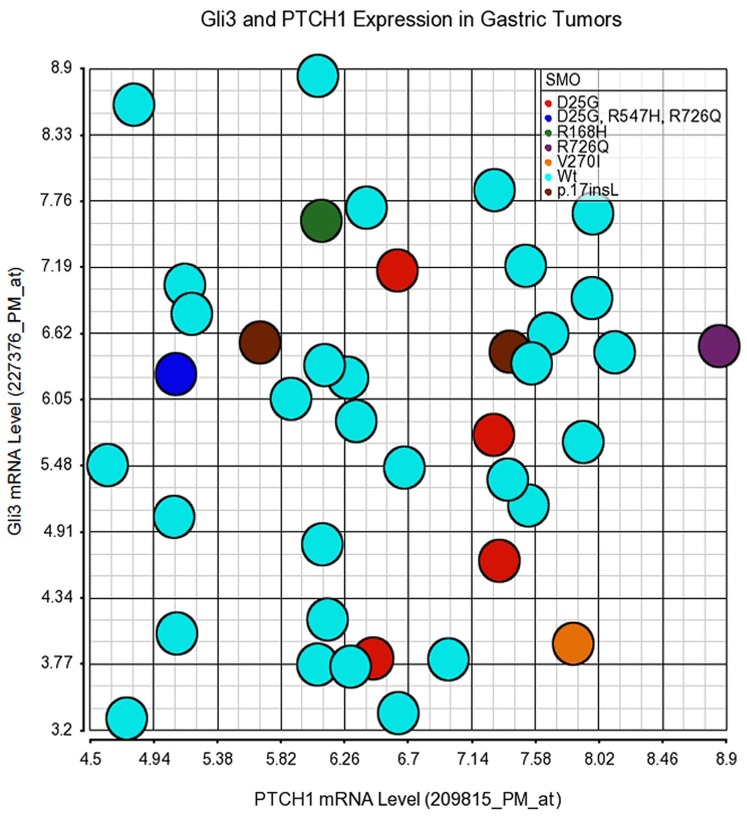
Expression of Gli3 and PTCH1 mRNA in gastric tumors with *SMO* mutations. Scatter plots for Gli3 and PTCH1, two hedgehog pathway down-stream genes expressed at relatively higher levels are shown according to their *SMO* mutations. Expression values have been RMA normalized and presented as log value. Note that except for the tumor with R726Q SMO mutation, all other tumors showed levels of Gli3 and/or PTCH1 within the range of tumors with wild type *SMO* gene.

**Figure 5:**
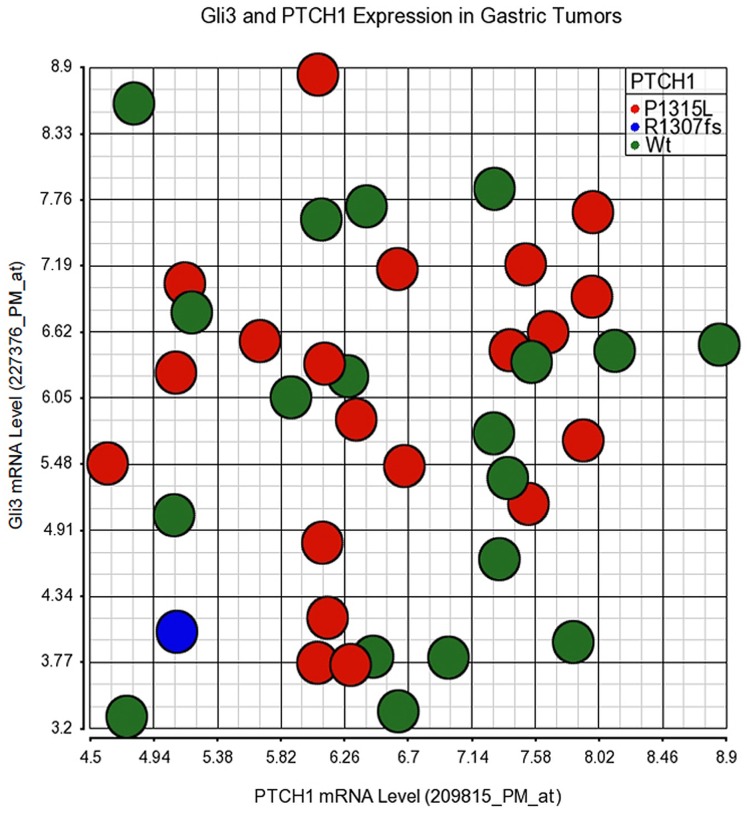
Expression of Gli3 and PTCH1 mRNA in gastric tumors with *PTCH1* mutations. Scatter plots for Gli3 and PTCH1, two hedgehog pathway down-stream genes expressed at relatively higher levels are shown according to their *PTCH1* mutation status. Expression values have been RMA normalized and presented as log value. Note the relative lower level of Gli3 and PTCH1 expression in the tumor with R1307fs mutation.

**Figure 6:**
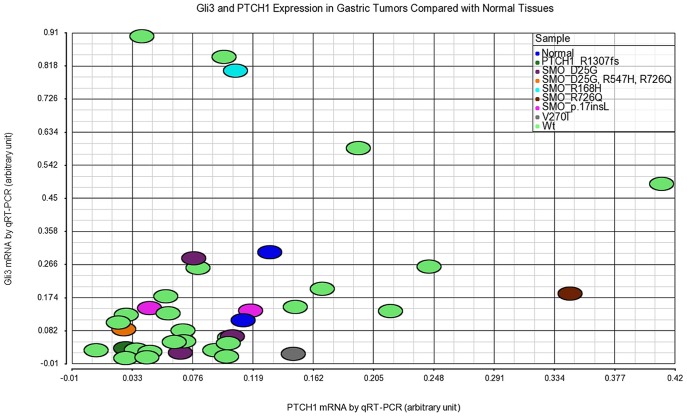
Expression of Gli3 and PTCH1 in gastric tumors compared with normal tissues examined by qRT-PCR. While some tumors expressed higher levels of Gli3 and PTCH1 than normal tissues, the overall expression levels of Gli3 and PTCH1 in tumor tissues, with or without SMO or PTCH1 mutations, are not markedly different from the normal tissues.

To gain insights into the potential impact of these novel pathway mutations on hedgehog pathway activity in tumors, the mRNA expression levels of hedgehog pathway responsive genes including *Gli1*, *Gli2*, *Gli3*, *PTCH1*, *PTCH2*, *SHH* and *IHH* were determined by microarray analysis using the corresponding freshly frozen tumor samples. Even with high RNA quality and adherence to stringent microarray quality control criteria (data not shown), the expression of hedgehog pathway genes such as Gli1 and Gli2 appear relatively low, with intensity (∼50) in the range of microarray detection limit. Gli3 and PTCH1 mRNA levels were relatively higher than other genes. The scatter plots illustrating the expression profiles of these two genes are shown in Fig. 4 and 5. When compared to the tumor populations with wild type *SMO* (Fig. 4) or wild type *PTCH1* (Fig. 5) genes, only the tumor with the R726Q SMO mutation, or that with the R1307fs *PTCH1* mutation, appears distinguishable from the majority of wild type tumors. The R726Q SMO mutant tumor expressed relatively high level of *PTCH1* and the R1307fs PTCH1 mutant tumor expressed relatively low level of both *PTCH1* and *Gli3*. It is noted that the R1307fs PTCH1 mutant tumor may have only 5–10% tumor components in the specimen as indicated by the sequencing traces, which may have contributed to the low expression levels of *PTCH1* and *Gli3*. The hedgehog pathway gene expression (Gli1, Gli2, Gli3, PTCH1, and PTCH2) in this set of tumors (35 of 39 had enough amount of total RNA) were also confirmed with real time quantitative RT-PCR. The data confirmed the microarray data showing higher expression levels of Gli3 and PTCH1 than other genes examined (not shown), and the relative expression levels of PTCH1 and Gli3 in samples with R726Q SMO or R1307fs PTCH1 as compared to other samples (Fig. 6). Two normal gastric tissues were also included in the qRT-PCR analysis. Overall, the tumors do not express dramatically higher levels of Gli3 and PTCH1 than normal tissues, although some tumors such as the ones with R726Q SMO and R168H SMO mutations do show higher expression than normal. Taken together, the mild or lack of down-stream gene overexpression suggests that the mutations identified in our study are probably non-driver mutations in gastric cancer.

The mutation frequencies in PTCH1 and SMO genes appear varied in different tumor types. In basal cell carcinoma, hedgehog activating mutations (either PTCH1 or SMO) occur in almost all tumors [3], [4], [5]. Earlier efforts with relative small numbers of tumors found mutations in subsets of medulloblastoma, breast cancer, and meningiomas, but not in colorectal or bladder carcinomas [19]. Maesawa et al. reported one nonsense mutation and one novel missense mutation in PTCH1 in 30 esophageal squamous cell carcinoma (7%) [20]. However, PTCH1 and/or SMO mutations are reportedly rare in chondrosarcoma [21] and trichoblastoma [22]. In our study, 5/39 (13%) tumors had novel mutations in either PTCH1 or SMO. Two recent studies utilizing next-generation sequencing approaches support our finding that PTCH1 or SMO are mutated at low frequency in gastric cancer. Sequencing of the coding regions of 384 cancer genes in 50 adenocarcinoma samples revealed only 2 potential loss of function mutations in PTCH1 (4%) [23], while no hedgehog pathway mutations were detected following exome sequencing of 22 gastric tumor samples [24]. It is interesting to note that in our study mutation frequency of SMO appears higher than that of PTCH1, while only PTCH1 mutations were found in the earlier study [24]. This on one hand suggests the non-random nature of these mutations, on the other hand may suggest that mutation profile varies in different patient populations.

Ascertaining the functional status of a mutation found in a tumor can be a significant challenge. In our study, we identified potential germline SNPs, confirmed the presence of the mutations in relevant tumor types, and examined the effect of the mutations on down-stream gene expression. We also recognized that assessing function of mutant genes based simply on mRNA levels may be confounded by a number of factors. Multiple levels of gene regulation, such as a gene product acting as both transcription activator and repressor (e.g., Gli3 vs Gli3R), and the presence of negative feedback loops (e.g. PTCH1), as well as the varied tumor content within the different tissue specimens all contributed to the complexity. Generation of appropriate cell line pairs expressing mutant versus wild type genes would potentially allow for a more conclusive functional assessment. However this approach has its own risk as in vitro propagated cells may not have demonstrable autocrine hedgehog signaling as reported [25], [26].

In summary, our data indicate that *SMO* and/or *PTCH1* mutations are present at low frequency in different histologic subtypes of gastric tumors and these however likely represent non-driver mutations.

## Supporting Information

Table S1 **Primers used for sequencing of** ***SMO*** **and** ***PTCH1*** **genes.**
(DOCX)
