# Supplementary material for: Mutations in the Hedgehog Pathway Genes SMO and PTCH1 in Human Gastric Tumors
Source: PLoS One. 2013 Jan 18;8(1):e54415. doi: 10.1371/journal.pone.0054415 (PMC3548780; doi:10.1371/journal.pone.0054415)
Supplement: Table S1 — Primers used for sequencing of SMO and PTCH1 genes. (DOCX) [file pone.0054415.s001.docx]

Table S1. Primers used for sequencing of *SMO* and *PTCH1* genes

| *SMO* | | | |
| --- | --- | --- | --- |
| Primer Name | Nucleotide Sequence | Primer Name | Nucleotide Sequence |
| SMO-1F | GGGGCTTTTGCTGAGTTG | SMO-1R | CCTGAGCCTGTTCTTTCCAA |
| SMO-2F | AAGAACTGTCCTGCCCAGATG | SMO-2R | CCACTGGACCCTGCCCTATAC |
| SMO-3F | AATAATTTGCCAAGCCAGCC | SMO-3R | CTTCTGATCATGACCCTTCCC |
| SMO-4F | AGGGTCATGATCAGAAGGGTC | SMO-4R | AGTATGCAGTAGGGCAGAGCC |
| SMO-5F | CTGACTTCTGGGAACCTCCAG | SMO-5R | GACAGAAGGTGGGTTACTGGC |
| SMO-6F | GTGGCGCAGGTATAGTGACTG | SMO-6R | GCCCTATAGGAGCTAGCTGGG |
| SMO-7F | GACTCCAGAGCCTTAGGACCC | SMO-7R | TCCCTATGGCTAACTTGTCCC |
| SMO-8F | AAGCAGTTCTTGGACTGAGCC | SMO-8R | CCATCCATTGAATCTGCTGTC |
| SMO-9F | AGTTGGAAGCTGCAGTGGG | SMO-9R | CAAGGCTGTGCTAGAGGCAG |
| SMO-10F | CTCTGGAAAGAATGGCATCG | SMO-10R | TTCCAAATAATCTGTGTGCCC |
| SMO-11F | AATGGCACTGACTATGGGAGG | SMO-11R | CCACTCTTCAGATCCTCTGGG |
| SMO-12F | AACAGGTTAAGTGCTCCCAGG | SMO-12R | CATGCTCGGTGAGGAAGAAG |
|  | | | |
| *PTCH1* | | | |
| Primer Name | Nucleotide Sequence | Primer Name | Nucleotide Sequence |
| PTCH-1F | GCTGGTCTGTCAACCGGAG | PTCH-1R | GTGTTTGTGTGTGGCGGG |
|  |  | PTCH-1SeqR | caaagagttagaggagggaag |
| PTCH-2F | AGTCTCGAGGGCGAGTCC | PTCH-2R | GCGCTGGCGAATATCTCTATC |
| PTCH-3F | GCTCACACATCAGCCAGTCTC | PTCH-3R | GCCTAAACCAGCAGCCTTC |
| PTCH-4F | AAGCTTGCTGGGTCTCTACTTG | PTCH-4R | GAGGCCATGCGTTAGGTTAAG |
| PTCH-5F | AGAAACAGGTTACATGGATCAGG | PTCH-5R | TGAATGAAATTTAATGACGCCTAC |
| PTCH-6F | GCGCAGCCGTGTTACTTTAC | PTCH-6R | AGGCTAATGGGAGGTGTATGG |
| PTCH-7F | CTCTCTGAAACACACAAGCCC | PTCH-7R | TACACTTGCCGATGTCAGGAG |
| PTCH-8F | AGAATTGCAGCCAGTGAGTTG | PTCH-8R | TCATCCCATCAAGTTCCCAG |
| PTCH-9F | CTGAGATCTGTGCTGTCGAGG | PTCH-9R | ACGCTCTCTCTGTCCTGGATG |
| PTCH-10F | ATGGGTGGAGGGAAACATTAG | PTCH-10R | AGGACACACAGCACACAGGAG |
| PTCH-11F | TGCTTCAGGAGCTGTTAGGTG | PTCH-11R | TGACACATCATCTGACATGGG |
| PTCH-12F | CTCTGTTTCCCTAATGCCAGC | PTCH-12R | AGCCTCAAACACAGGCATTTC |
| PTCH-13F | TGTCACGGTTTCAAATGCTTC | PTCH-13R | GTTCTCCACACCAGCACAAAC |
| PTCH-14F1 | GATGTTATCAACCAGGCGATG | PTCH-14R1 | GTTGTGGCAGATTACCTTGGC |
| PTCH-14F2 | GGTTGAACCTCAGGCCTACAC | PTCH-14R2 | ACTCCCATGGAAGATGACCTC |
|  |  | PTCH-14R2-Seq | gaaaaagaagaaaagtagaagc |
| PTCH-15F | GATAAATCAGTTTAAGTGTGGTGGTG | PTCH-15R | TCATAATCATGACAAAGGAACCTG |
| PTCH-16F | TTCAGTCAAAGTGGATGTGGG | PTCH-16R | CTTTCTACCAGCTCCCAGTGC |
| PTCH-17F | TGCTCTCAAGGCAGAAGTGTG | PTCH-17R | TGAAGGCTGTTGCTGAGTTTG |
| PTCH-18F | GAGGCTATGATCAGCATTGTTTG | PTCH-18R | ACTTCCCGGCTGCAGAAAG |
| PTCH-19F | CTGAACCGAGGACACCTTAGC | PTCH-19R | GGTTCCCACTTGGAGACAAAC |
| PTCH-20F | TGAGCAGTTCTGAGAGCTTGTAAC | PTCH-20R | TCCTTGACCTTCTGATCCACC |
| PTCH-21F | TGTGAACTGCGGTTGGATAAC | PTCH-21R | AGTATCGAAGTGAAGAGCGGC |
| PTCH-22F | TACCGTGCTTTGAGCTTTGAG | PTCH-22R | CCATCTGCCTGTGTGATGTG |
| PTCH-23F1 | AACCCAAGGAGGGAAGTGTG | PTCH-23R1 | CTGCAGCTCAATGACTTCCAC |
| PTCH-23F2 | GCATTCTGGCCCTAGCAATAG | PTCH-23R2 | AGCTTGGACACATCAGCCTTG |
